# An Evolutionary Preamble Towards a Multilevel Framework to Understand Adolescent Mental Health: An International Delphi Study

**DOI:** 10.3390/children12091189

**Published:** 2025-09-05

**Authors:** Federica Sancassiani, Vanessa Barrui, Fabrizio Bert, Sara Carucci, Fatma Charfi, Giulia Cossu, Arne Holte, Jutta Lindert, Simone Marchini, Alessandra Perra, Samantha Pinna, Antonio Egidio Nardi, Alessandra Scano, Cesar A. Soutullo, Massimo Tusconi, Diego Primavera

**Affiliations:** 1 Department of Medical Sciences and Public Health, University of Cagliari, 09042 Monserrato, Italy; federica.sancassiani@unica.it (F.S.); vanessa.barrui@aslogliastra.it (V.B.); sara.carucci@unica.it (S.C.); giulia.cossu@unica.it (G.C.); alessandra.perra@unica.it (A.P.); samantha.pinna@unica.it (S.P.); diego.primavera@unica.it (D.P.); 2 Child and Adolescent Neuropsichiatry Unit, “A. Cao” Pediatric Hospital, P.O. Microcitemico, ASL Cagliari, 09121 Cagliari, Italy; 3 Department of Public Health and Pediatrics Sciences, University of Turin, 10124 Turin, Italy; fabrizio.bert@unito.it; 4 Research Laboratory LR22SP01, Department of Child Psychiatry, Mongi Slim Hospital, Faculty of Medicine of Tunis, University of Tunis El Manar, Tunis 1068, Tunisia; fatma.charfi@fmt.utm.tn; 5 Department of Psychology, University of Oslo, 0313 Oslo, Norway; arne.holte@psykologi.uio.no; 6 Department of Social Work and Health, University of Emden/Leer, 26723 Emden, Germany; jutta.lindert@hs-emden-leer.de; 7 Department of Child and Adolescent Psychiatry, Site Anderlecht, Hôpital Universitaire de Bruxelles, 1070 Brussels, Belgium; simone.marchini@ulb.be; 8 Laboratory of Developmental Psychiatry, Faculty of Medicine, Université Libre de Bruxelles, 1070 Brussels, Belgium; 9 Laboratory of Panic and Respiration-IPUB, Institute of Psychiatry of the Federal University of Rio de Janeiro, Rio de Janeiro 22290-140, Brazil; antonio.nardi@ipub.ufrj.br; 10 Department of Surgical Sciences, University of Cagliari, 09042 Monserrato, Italy; 11 “Louis A. Faillace MD” Department of Psychiatry and Behavioral Sciences, McGovern Medical School, The University of Texas Health Science Center at Houston (UTHealth Houston), Houston, TX 77054, USA; cesar.a.soutullo@uth.tmc.edu; 12 Center of Liason Psychiatry and Psychosomatics, University Hospital of Cagliari, 09126 Cagliari, Italy; m.tusconi@aoucagliari.it

**Keywords:** Delphi technique, adolescent, mental health, social determinants of health, epigenesis, genetic

## Abstract

Background/Objectives: Adolescence is a sensitive developmental window shaped by both vulnerabilities and adaptive potential. From an evolutionary standpoint, mental health difficulties in this period may represent functional responses to environmental stressors rather than mere dysfunctions. Despite increasing interest, integrative models capturing the dynamic interplay of risk and protective factors in adolescent mental health remain limited. This study presents a holistic, multi-level framework grounded in ecological and evolutionary theories to improve understanding and intervention strategies. Methods: A two-round Delphi method was used to develop and validate the framework. Twelve experts in adolescent mental health evaluated a preliminary draft derived from the literature. In Round 1, 12 items were rated across five criteria (YES/NO format), with feedback provided when consensus thresholds were not met. Revisions were made using consensus index scores. In Round 2, the revised draft was assessed across eight broader dimensions. A consensus threshold of 0.75 was used in both rounds. Results: Twelve out of thirteen experts (92%) agreed to join the panel. Round 1 item scores ranged from 0.72 to 0.85, with an average consensus index of 0.78. In Round 2, ratings improved significantly, ranging from 0.82 to 1.0, with an average of 0.95. The Steering Committee incorporated expert feedback by refining the structure, deepening content, updating sources, and clarifying key components. Conclusions: The final framework allows for the clustering of indicators across macro-, medium-, and micro-level domains. It offers a robust foundation for future research and the development of targeted, evolutionarily informed mental health interventions for adolescents.

## 1. Introduction

### 1.1. The Weight of the Issue

The impact of mental health-related problems during adolescence is profound. The challenges faced during this critical developmental stage can significantly shape and compromise wellbeing across the lifespan [1,2,3]. Adolescence is a critical period during which young individuals transition from the protective and reassuring identification with parental figures to explore the enticing yet challenging world of autonomy. This exhilarating but precarious phase has been described as “the divided adolescent self” [4]. To navigate this stage successfully, adolescents must develop new capabilities across several domains:

- Biological skills: Hormonal, sexual, and cerebral maturation processes.
- Cognitive skills: The emergence of abstract thinking, advanced reasoning and a stable sense of identity.
- Emotional skills: Differentiation, recognition, and self-regulation of emotions.
- Social skills: The ability to decentrate and manage social roles within (peer) groups.

The criticality of this phase and its consequences on the subsequent life path are well illustrated by some statistics about mental health problems in this phase of life: 5–7% of adolescents experience a mood disorder, with higher prevalence in females [5]. Other prevalent conditions in this population include ADHD, eating disorders, personality disorders, anxiety disorders, and substance abuse [5,6,7].

Many mental disorders first manifest during developmental age, with a peak incidence around 14.5 years old [8]. This allows for the identification of a temporal continuum that begins with phobias/separation anxiety/autism spectrum disorder/ADHD/social anxiety (8–13 years), followed by anorexia nervosa/bulimia nervosa/obsessive-compulsive/binge eating/cannabis use disorders (17–22 years), schizophrenia, personality, panic and alcohol use disorders (25–27 years), and finally post-traumatic/depressive/generalized anxiety/bipolar/acute and transient psychotic disorders (30–35 years), with overlap among groups and no significant clustering [8]. Furthermore, there is an age and sex dimension to consider. Before puberty, among those with mental disorders there is a predominance of boys, and neurodevelopmental disorders are the most common. After puberty, there is a predominance of girls, with depression, anxiety, and eating disorders being more prevalent.

Beyond specific psychiatric diagnoses, professionals working with adolescents frequently encounter issues such as self-harm. This behavior affects at least 10% of adolescents at some point in their lives, being more prevalent in females and during late adolescence [9].

Another devastating problem is suicide and attempted suicide [10,11]. Suicide attempts impact approximately 2.5% of adolescents, with significant variability across cultural contexts [12,13,14]. Although lower in frequency compared to other age groups, suicide among adolescents is most prevalent in late adolescence and ranks as the second leading cause of death among individuals aged 10 to 24 in the United States [15].

If the intent is prevention, it is critical to note that 1 in 4 young people are dissatisfied with their lives. Major concerns include inability to cope with stress, academic or school-related problems, body image issues, family conflicts, bullying and cyberbullying, difficulties in social interactions and external judgment, exemplified by the pervasive belief, “If I’m not perfect, then I’m worthless” [16,17].

Three main factors appear to increase the risk of suicidality among adolescents [18]: psychological factors (depression, anxiety, previous suicide attempt, drug and alcohol use, and other comorbid psychiatric disorders), stressful life events (family problems, peer conflicts, child abuse, academic stressor), and personality traits (such as neuroticism, impulsivity, low self-esteem).

### 1.2. Limitations of Current Approaches on Adolescents’ Mental Health

Conventional clinical frameworks on adolescents’ mental health have focused on identifying distinct mental disorders, primarily as a foundation for guiding evidence-based treatment decisions. However, these “distinct” disorders often reflect fully developed, prototypical, and relatively advanced-stage syndromes typically addressed within specialized adult or secondary mental health services globally [19]. Acknowledging the limitations of current psychiatric classification and treatment frameworks, particularly in addressing the needs of young individuals and supporting early intervention services, transdiagnostic clinical staging models and dimensional approaches have emerged as more adequate alternatives [19,20].

These models, mainly focusing on the vulnerability to stress, aim to identify factors linked to the emergence of symptoms [21,22] and mental health-related difficulties. They illustrate the relationship between stress and the onset of (psycho)pathology, suggesting an interaction among (1) latent endogenous vulnerability factors that, when combined with stress, exacerbate the negative effects of stressful conditions, (2) environmental factors that affect the onset and progression of (psycho)pathology, and (3) salutogene and protective factors that buffer or lessen the impact of stress on pathological outcomes.

Extensive research [23] has explored risk factors for adolescents’ mental health across various dimensions, including genetics, individual psychological processes, neurocognitive development, local environment (i.e., substances of abuse, bullying, family dynamics, neglect and childhood trauma, poor academic progression, school failure, school avoidance, household economy, degree of poverty, crime, income level, density of social networks, neighbourhood quality, quality of buildings, shared culture and values, etc.). On the other hand, research on developmental resilience has pointed out that there may be several resilience factors that provide transdiagnostic protection against the effects of adverse experiences on the risk for psychopathology and promote mental health and positive development in general and among adolescents at risk for psychopathology [24]. The interrelationship between macro-social factors and adolescents’ mental health outcomes has garnered increasing attention. Here, the role of social support networks cannot be understated. Supportive relationships with family, peers, and mentors can buffer against the negative effects of social inequalities. Young individuals with strong support systems often exhibit better emotional resilience and coping mechanisms, which can enhance their overall psychological health [25]. At the individual level, physical activity [26,27], a healthy diet [28,29], and regular sleeping habits [30] also represent important protective factors for adolescents’ mental health.

Despite the amount of research, significant gaps remain in providing integrative frameworks capable of capturing the interplay between risk and protective factors in adolescents’ mental health from an evolutionary perspective [31,32]. Adolescence represents a transitional period in which young individuals face significant challenges related to physical, cognitive, and emotional growth, and these challenges are partly the result of evolutionary pressures that promote survival and reproduction. While these changes are normative, they can increase vulnerability to mental health issues, but they can also foster the development of adaptive skills when supported by positive environments and healthy relationships. Adolescent mental health difficulties should not be viewed solely as dysfunctions but also as adaptations that reflect responses to evolutionary challenges. Furthermore, the ecological model of health emphasizes the necessity of considering multiple layers of influence, from individual factors to broader social policies [33].

Future research should adopt a holistic, multi-level perspective that integrates all these dimensions. Such an approach requires collaboration across disciplines to unravel the complex interplay between genetic, individual psychological, neurocognitive, micro-, medium and macro environmental factors affecting adolescent mental health. By understanding these relationships, interventions can be designed to address the root causes of mental health issues among youth, promoting healthier development and well-being.

### 1.3. Proposed Focal Domains and Aims

To overcome the limitations previously described, we propose five key areas of focus as the basis for conceptualizing an integrative and multi-level framework on adolescent mental health. These focal domains, identified through a critical appraisal of the literature, provided the foundation for the consensus-building process conducted using the Delphi methodology. Each includes several factors of vulnerability or protection that are supported by transdisciplinary evidence:

- The “social” brain during adolescence: Neuroscientific literature highlights adolescence as a period of heightened neuroplasticity in socio-emotional brain circuits. Social experiences, including peer interactions, acceptance, and rejection, play a crucial role in shaping neural development, identity formation, and emotional regulation during this phase.
- Social determinants, macro-social and environmental factors: As underscored by ecological models, adolescents are particularly exposed to structural risk factors such as poverty, housing insecurity, neighborhood violence, and inequality, all of which have enduring effects on mental health trajectories.
- Discrimination, social stigma, and adolescents’ beliefs about mental health: Stigma-related barriers often deter help-seeking behaviors and shape internalized beliefs, particularly among youth with marginalized identities. This area addresses the need to counteract social exclusion and to understand how beliefs and values about mental illness develop and impact behavior.
- Online behaviors, socialization, and loneliness: Digital environments profoundly influence peer interactions, identity negotiation, and exposure to cyberbullying or unrealistic social comparisons. Adolescents’ mental health is increasingly shaped by online dynamics, which can amplify both risk and protective processes.
- Pre-clinical conditions: Many disorders emerge gradually, with prodromal symptoms or non-specific psychological distress preceding clinical onset. A developmental and evolutionary lens allows us to capture these transitional states and informs transdiagnostic clinical staging models aimed at early prevention.

**Figure 1:**
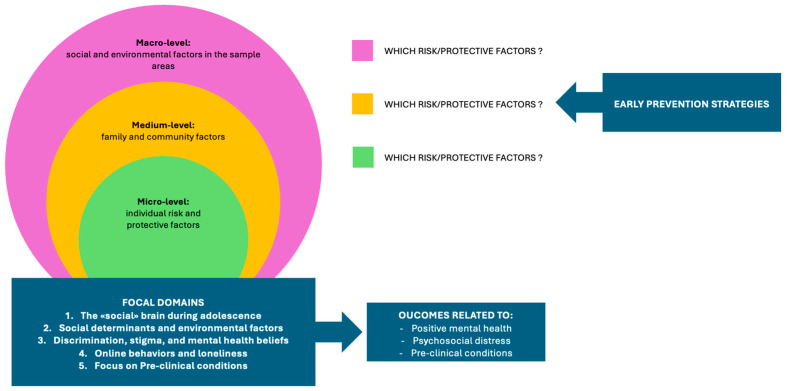
Developing a Multilevel Framework for Adolescent Mental Health.

Considering these domains, the present Delphi study aimed to examine, discuss and establish a consensus among a panel of Experts from diverse backgrounds on risk and protective factors at the individual (micro), family and community (medium), and socio-environmental (macro) levels that can influence positive mental health, psychosocial distress, and pre-clinical conditions during adolescence (see Figure 1). This evidence-informed consensus was intended to shape a framework capable of underpinning explanatory models of adolescent mental health and inform early prevention strategies.

## 2. Materials and Methods

A two-round Delphi methodology was applied to develop and define the framework on adolescents’ mental health and the related draft. The Delphi method is a structured approach designed to systematically achieve expert consensus on a specific research topic through iterative surveys [34]. Panel members (usually seven or more) provide anonymous feedback and refine their responses based on aggregated group input. This iterative process continues until consensus or response stability is attained. The Delphi methodology is widely employed in disciplines that rely on expert judgment to develop and validate novel contents (i.e., guidelines, theoretical frameworks, evaluation tools).

Following the Delphi methodology, this study was designed and implemented as follows: (1) selection and invitation of an expert panel; (2) provision of detailed information to the panel regarding the research question and subject matter; (3) execution of two Delphi rounds to achieve consensus on predefined items. The research team included 2 psychiatrists, 1 health psychologist, 1 psychotherapist, 1 sociologist, 1 technician in psychiatric rehabilitation. It worked as a Steering Committee to develop the initial version of the multidimensional framework on adolescents’ mental health and the resulting draft, carry out the execution of Delphi rounds, and conduct the subsequent data analysis.

### 2.1. Description of the First Draft of the Framework

Based on a critical literature overview, the Steering Committee proposed and discussed five focal domains to address some gaps in the evidence about adolescents’ mental health. Domains where developmental sensitivity, ecological relevance, and emerging epidemiological trends converge to shape mental health outcomes during adolescence were prioritized. Then, the Steering Committee drafted an initial version of the multidimensional framework. This version included a title and eleven paragraphs with bibliographic references. Paragraphs 1 and 2 addressed the background, specifically the weight of the issue and the limitations of current approaches to adolescent mental health. Paragraphs 3, 4, 5, and 6 focused on the first focal domain, namely “The “social” brain during adolescence”, with particular attention to brain development, the interaction between genetic and environmental factors in neurodevelopment, the evolutionary advantages of social bonds and networks, and the relationship between epigenetics and attachment styles. Paragraph 7 addressed the second focal domain, namely “Social determinants, macro-social and environmental factors”. Paragraph 8 was dedicated to the third focal domain on “Discrimination, social stigma, and adolescents’ beliefs about mental health”. Paragraph 9 covered the fourth focal domain, focusing on “Online behaviors, socialization, and loneliness”, while paragraph 10 dealt with “Pre-clinical conditions”, particularly the opportunity to frame certain prodromal manifestations from a developmental and evolutionary perspective. Finally, paragraph 11 proposed applying the shared conceptual framework, centered on the five focal domains, within a multi-level matrix of indicators/determinants of adolescent mental health (see Table 1).

This initial draft was used as a basis for the current Delphi study, to be submitted to the expert panel validation and refined iteratively.

### 2.2. Panel Selection

A panel of experts specializing in Child Neuropsychiatry, Psychiatry, Psychology, Sociology, Public Health, Molecular Medicine and Genetics were invited to participate. The selection was conducted through the clinical and research network of the research team, based on high-quality publications in the field of adolescents’ mental health. This approach aimed to ensure a diverse, international, and representative expert panel.

### 2.3. Consensus Process

This Delphi study included two separate rounds to reach a consensus. The rounds were conducted via emails sent separately to each panel member, allowing them to express their opinions independently. Specific forms were designed, including instructions for performing the rounds and systematically collecting their contributions.

In the first round, the panel’s members were asked to evaluate five categories (relevance, consistency, clarity, ease of understanding, and updating) about 12 items (the title and each of the eleven paragraphs of the initial draft version). They could respond with YES or NO for each category for each item. If they answered NO in three or more categories, they were given space to write comments and propose improvements to the title or paragraph under review, supported by bibliographic references. Once all panel members’ forms were collected, the research team calculated consensus indexes and made all necessary modifications to the draft, strictly adhering to the panel’s feedback and explicitly detailing the methodology used to develop the revised version of the draft.

In the second round, the panel’s members were asked to evaluate eight categories (relevance, consistency, clarity, ease of understanding, updating, consistency with suggested changes, improvement compared with the initial draft, and suitability for publication of one item (the entire corrected version of the draft). Each panel member could respond with YES or NO for each category and comment on their responses.

The categories for item evaluation during the first round can be described as follows:

- Relevance: The degree to which the item aligns with the research objectives and contributes meaningfully to the topic under investigation.
- Consistency: The extent to which the item maintains logical coherence and aligns with the overall structure of the research.
- Clarity: The item is well-defined, unambiguous, and structured coherently, without contradictions or confusing elements.
- Ease of understanding: The item is accessible in terms of language complexity and comprehensibility for readers with varying levels of expertise.
- Updating: The extent to which the item reflects the most current evidence, theories, and advancements in the field.
- In the second round, in addition to the five categories used in the first round, the following were added:
- Consistency with suggested changes: The extent to which the item incorporates and aligns with the modifications proposed in the previous round.
- Improvement compared with the initial draft: The degree to which the item demonstrates enhancements in clarity, coherence, and overall quality relative to the original version.
- Suitability for publication: The item’s appropriateness for dissemination, considering its scientific rigor, completeness, and adherence to academic standards.

Each YES and NO response was assigned 1 and 0 points in both rounds, respectively. In the first round, the maximum overall score for consensus about the items was 720 (each of the 12 items was evaluated on five categories by 12 members, and each category could have a maximum score of 1: 12 × 5 × 12 = 720). In the second round, the maximum overall score for consensus about the items was 88 (the item was evaluated on eight categories by 11 members, and each category could have a maximum score of 1: 1 × 8 × 11 = 88).

In both rounds, the average consensus index was calculated for the two versions of the draft (dividing the actual overall score for consensus by the maximum overall score for consensus). The recommended consensus index cut-off value of 0.75 was considered acceptable [35,36].

Following the first round’s results, in addition to the consensus index, the research team considered all cases in which a panel member assigned at least three NO responses to a given item and suggested specific modifications to the draft. In these instances, all recommendations were accepted and integrated with those provided by other panel members. As a result, the initial version of the draft underwent several modifications, some of which were substantial, before proceeding to the second round.

To ensure clarity and ease of completion for all panel members, the Delphi process was deliberately based on a dichotomous (YES/NO) evaluation format. This choice was intended to maximize objectivity and comparability across all items and categories, and to reduce variability in interpretation due to differing professional backgrounds. This approach allowed us to identify which areas were deemed acceptable or in need of revision.

Based on the results of the second round, in addition to considering the consensus index, the research team reviewed all cases in which individual panel members proposed further revisions to the draft. In such cases, all suggestions were accepted and integrated alongside those provided by other panel members to finalize the definitive version of the draft.

## 3. Results

### 3.1. Panel Composition

Initially, 13 experts were contacted from the research team’s clinical and research network. Among them, 12 (92%) agreed to join the panel. Based on the backgrounds of its members, the panel includes 6 child and adolescent neuropsychiatrists, 2 psychiatrists, 2 specialists in public mental health, 1 specialist in public health, and 1 molecular biologist and geneticist. Based on the members’ country of origin and/or academic affiliation, 5 were from Italy, 1 from Tunisia, 1 from Norway, 1 from Germany, 1 from Belgium, 1 from Brazil, 1 from the United States, and 1 from Albania.

### 3.2. Delphi Process

#### 3.2.1. Round 1

All the 12 members of the panel participated in the first round. Table 2 summarizes the YES/NO responses from panel members during the first round. As reported, five members provided YES responses for all categories across all items. One member provided YES responses for all items except for a single NO across the five categories related to the title. Six members assigned at least three NO responses across the five categories for at least one item. Modifications were made to the first version of the draft based on their recommendations.

Table 3 reports the consensus assessment among the panel members during the first round. If all twelve members agreed on an item’s consistency, for instance, it received a score of 12 out of 12. Each item had a maximum possible score of 60.

When evaluated for the performance of the items, the score range for each item was 0.72–0.85 (43/60–51/60). The overall score was 565/720, yielding an average consensus index of 0.78. These results suggest that the first version of the draft was generally relevant (0.86), clear (0.75), and easy to understand (0.86). Notably, the fifth paragraph (0.72), as well as the categories “Consistency” (0.71) and “Updating” (0.74), did not reach the recommended minimum consensus threshold (0.75). Therefore, in revising the first version of the draft, the research team focused particularly on these aspects.

The revised and corrected draft adopted the editorial format of a scientific article, serving as the basis for the second round of the Delphi process. In addition to the background, the objective was explicitly stated, the Delphi methodology adopted was described in detail, and the results of both rounds were reported and discussed in this version. These findings enabled the definition of a multi-level framework on adolescent mental health.

#### 3.2.2. Round 2

One panel member did not participate in the second round; therefore, responses were collected from 11 members. Table 4 summarizes the YES/NO responses from all 11 panel members during the second round. As reported, nine members provided YES responses for all categories. One member provided YES responses for all items except for a single NO across the eight categories related to the suitability for publication. One member assigned YES responses for all items except for two NO answers related to updating and suitability for publication. Four members provided comments on the text, suggesting minor revisions, and modifications were made to the draft based on their recommendations.

Table 5 reports the consensus assessment among the panel members during the second round. If all eleven members agreed on an item’s consistency, for instance, it received a score of 11 out of 11. Each item had a maximum possible score of 88.

The item considered for the second round was the revised version of the draft following the first round. The overall score was 84/88, yielding an average consensus index of 0.95. The range of scores assigned by the panel members was 0.82/1.0 (9/11–11/11). The recommended minimum consensus threshold (0.75) was reached in all the categories. The results suggest that the new version of the draft after Round 1 was strongly relevant (1.0), consistent (1.0), clear (1.0), easy to understand (1.0), improved when compared to the first version (1.0), consistent (0.91), updating (0.91) and suitable to be published (0.82).

#### 3.2.3. Development of the Framework on Adolescents’ Mental Health

Through the two Delphi rounds, panel members evaluated the five focal domains proposed for the framework development and the draft in general. Following the first round, the Steering Committee implemented a series of modifications, carefully incorporating all the comments, suggestions, revisions, and integrations proposed by the panel members.

The main modifications involved a more coherent and precise distribution of the different sections according to a Delphi study, the revision of outdated or less relevant bibliographic sources, the selection of more impactful titles for the various paragraphs and sub-paragraphs, and a more in-depth development of certain parts that were only superficially addressed in the first draft.

The framework was developed based on the systematization of five focal domains proposed by the Steering Committee to address gaps in the existing literature, incorporating the panel’s recommendations for integration and further elaboration following the first and second rounds. Table 6 provides a summary of the main revisions from the initial draft version (see also Table 1 for a comparison). 

**Figure 2:**
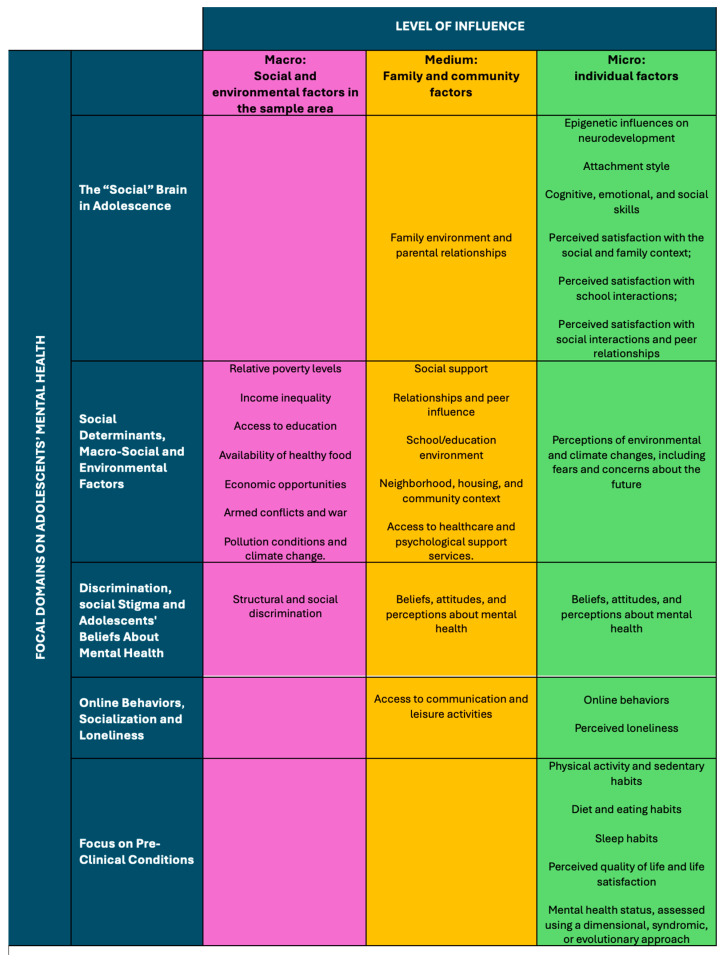
From a Multi-Level Framework to a Matrix of Measures for Research on adolescents’ mental health. Developed from five focal domains, the framework integrates macro-level (social and environmental), medium-level (family and community), and individual-level (adolescent) factors, delineating modifiable targets for early intervention and prevention. Its multilevel orientation supports integrative strategies across healthcare, education, and social sectors. The factors presented within the cells were identified and discussed through the Delphi consensus process. They are illustrative rather than exhaustive, and both the domains of interest and other components of the framework may be refined over time.

Figure 2 represents the final version of the integrative and multi-level framework, with a matrix of measures for research on adolescents’ mental health. Compared to Figure 1, this figure presents the risk and protective factors for each layer (micro, medium, macro) identified and refined through the iterative, updated literature review on the five focal domains of adolescent mental health conducted during the Delphi process.

At the individual level (micro), the identified risk and protective factors include: epigenetic influences on neurodevelopment; attachment style; cognitive, emotional, and social skills; physical activity and sedentary habits; diet and eating habits; sleep habits; perceived satisfaction with the social and family context; perceived satisfaction with school interactions; perceived satisfaction with social interactions and peer relationships; perceptions of environmental and climate changes, including fears and concerns about the future; online behaviors; perceived loneliness; perceived quality of life and life satisfaction; beliefs, attitudes, and perceptions about mental health; and mental health status, assessed using a dimensional, syndromic, or evolutionary approach.

At the family and community level (medium), the identified risk and protective factors include family environment and parental relationships; social support; relationships and peer influence; school/education environment; neighborhood, housing, and community context; access to communication and leisure activities; and access to healthcare and psychological support services.

At the social and environmental level (macro), the identified risk and protective factors include relative poverty levels; income inequality; access to education; availability of healthy food; economic opportunities; structural and social discrimination; armed conflicts and war; pollution conditions; and climate change.

## 4. Discussion

The integrative and multilevel framework on adolescents’ mental health proposed in this study underwent substantial revision through a two-round Delphi process, which resulted in both structural and content-related improvements from an initial draft. Key changes from the Experts’ panel included the explicit clarification of the study design and international scope, the expansion and updating of epidemiological and theoretical sections, and the specification of the study’s objective and focal domains regarding adolescents’ mental health. Furthermore, methodological sections were refined to detail the consensus process, and a clearer operationalization of the framework was introduced through a multi-level matrix of measures. Across both rounds, particular attention was given to strengthening the conceptual foundations of the five focal domains regarding adolescents’ mental health, ranging from neurodevelopmental aspects to socio-environmental determinants and pre-clinical conditions. This enabled the Experts’ panel to identify and discuss the key risk and protective factors influencing positive mental health, psychosocial distress, and pre-clinical conditions among adolescents. The following sections outline the results of these discussions, which underpin the development of the multi-level framework and its operationalization, with the dual purpose of advancing explanatory models of adolescent mental health and supporting the design of effective preventive strategies.

### 4.1. Focal Point 1: The “Social” Brain in Adolescence

#### 4.1.1. Brain Development

The human brain undergoes significant maturation into the second and third decades of life [23]. Adolescent brain development is characterized by a reduction in cortical gray matter volume, alongside increases in white matter volume and density [23,37]. These structural changes facilitate long-range axonal myelination and enhanced neural connectivity. Concurrently, synaptic pruning occurs within neural circuits associated with social cognition and processing [38,39]. Such neurobiological changes underpin key developmental milestones, including acquiring abstract reasoning [40] and establishing an independent sense of self, often achieved through a dialectical and sometimes conflictual separation process from parental figures [41].

Particularly, neurobiological models emphasize asynchronous brain development during adolescence, and the need to consider the interplay between limbic subcortical regions and prefrontal top-down control systems [42]. These models highlight distinct developmental trajectories, with limbic systems maturing earlier than prefrontal control regions. As a result, adolescents are disproportionately influenced by the functionally mature limbic system, creating an imbalance relative to the less mature prefrontal control system. This contrasts with children, where both systems are still developing, and adults, where both systems are fully mature. This framework explains nonlinear behavioral changes during development, stemming from the earlier maturation of limbic regions compared to the delayed maturation of prefrontal control regions. Over time, with ongoing development and experiential learning, functional connectivity between these regions facilitates top-down regulatory mechanisms. Moreover, these models resolve the apparent contradiction between high rates of risky behavior in adolescence, as reflected in health statistics, and the cognitive capability of adolescents to reason and understand risks [42].

#### 4.1.2. Genes and Environmental Factors

A significant amount of research has investigated genetic influences on neurodevelopment and the interaction between genetic and environmental factors. It is relevant to identify genetic risk markers that explain how exposure to certain environmental factors is associated with a higher likelihood of developing a disorder or a more severe form of it. Additionally, it is crucial to highlight how some disorders with a strong genetic component can “shape” the environmental exposure of the patient. For instance, ADHD is associated with higher risks of accidents, increased chances of head injuries, or early sexual experiences [43]. Bipolar disorder is linked to increased risk-taking regarding financial and sexual behaviors, as well as the risk of losing a job or dropping out of school earlier [44]. In this manner, genes may contribute to determining the response to environmental exposure while also predisposing individuals to specific environmental exposures.

Other models often highlight early life experiences, such as exposure to trauma or perinatal infections, which can emerge in adolescence as cognitive or behavioral issues. For example, neurodevelopmental theories of schizophrenia [45] provide valuable insights into these intricate relationships.

Moreover, links have been established between early-life trauma or maltreatment, whether occurring perinatally or during childhood, and the later development of psychiatric disorders in adolescence or early adulthood. Such conditions encompass eating disorders [8,46], post-traumatic stress disorder (PTSD) [47], borderline personality disorder [48] and psychosomatic disorders like fibromyalgia [49]. Importantly, prolonged overactivation of pain and stress response systems may lead to hypersensitivity and reduced activation thresholds in central neural circuits [49].

Despite these advances, relatively few studies have investigated how external physical and social environments influence neurodevelopment during adolescence, leaving a critical gap in our understanding of these interactions.

#### 4.1.3. Social Bonds and Networks

Humans have historically adapted to their environments through complex networks of interaction. The evolutionary advantages of establishing social bonds and networks include improved access to essential resources such as food, protection from predators, reproductive opportunities, emotional support, and self-esteem enhancement [50]. Research on both humans and non-human primates highlights a strong correlation between social behaviors within peer networks and the complexity, size, and functionality of brain structures [51,52,53].

Recent research reveals that cognitive empathy mediates the relationship between the gray matter volume of the dorsomedial prefrontal cortex and the size of social networks. These findings highlight a bidirectional and dynamic relationship between social interactions and brain development during adolescence, emphasizing the critical interplay between environmental factors and neurodevelopmental processes [54].

High-status primates play a significant role in influencing the social roles and behaviors of lower-status individuals. Nonhuman primates with elevated social status, as determined by the frequency of dominance behaviors observed during ethological studies, exhibit greater functional connectivity between social brain regions in the frontal and temporal cortices compared to their lower-status counterparts [50]. These neurological traits enhance their ability to achieve and maintain dominance and high social status, even when introduced to a novel group [51]. This phenomenon suggests that humans with dominant or influential personality traits may similarly possess enhanced capacities for social success across diverse contexts [55]. As social networks grow more complex, mechanisms that promote alliances, rather than just dominance, become ever more essential.

#### 4.1.4. Attachment Styles and Epigenetics

Because most models of social dynamics derive from studies on nonhuman primates, it is essential to investigate how the capacity for alliance formation shapes human adolescent development. Gender differences in alliance mechanisms, in particular, warrant further exploration.

In humans, the epigenetic processes underpinning alliance formation are highly intricate, with attachment to primary caregivers serving as a foundational factor [56,57]. Attachment mechanisms, extensively studied in the context of distress responses and interpersonal strategies, were initially examined through ethological observations of mother-infant interactions conducted by John Bowlby’s research group [58,59]. Differences in attachment styles—secure, anxious/ambivalent, avoidant, and disorganized—remain central to understanding these processes.

Nonetheless, key aspects of the sociobiology of human adolescent groups remain underexplored. First, modern society is evolving rapidly, leading to swift and often unpredictable modifications in adaptive behaviors. This is especially critical during adolescence, a developmental period characterized by heightened sensitivity to change, exploration, and self-discovery. Second, and closely related to the first point, secure attachment may play a pivotal role in mediating behaviors that initially appear maladaptive but could become advantageous in an ever-changing social landscape.

The attachment theory has been reviewed extensively in light of adverse childhood events and their impacts on brain development [60,61,62,63,64]. This research offers an attempt to explain epigenetic modifications in genes associated with social stress regulation and hypothalamic–pituitary–adrenal (HPA) axis functioning. Furthermore, it contributes to highlighting that secure attachment is a significant predictor of well-being in youth, fostering enhanced resilience to adversity and greater adaptive functioning [65], as well as the critical role of insecure attachment styles in mediating the onset of mental health problems during key developmental stages. Although attachment styles are primarily defined in early childhood, they become especially relevant during adolescence, a period when individuals develop skills to establish their social roles. Consequently, studying attachment styles during this critical stage is essential to understanding how adolescents navigate the complexities of contemporary social environments.

### 4.2. Focal Point 2: Social Determinants, Macro-Social and Environmental Factors

The influence of social determinants on individual behavior and health has a long tradition [66]. Socioeconomic status (SES), and poverty [67], war, conflicts [68] and migrations [69], family environment and parental relationships [70], social relationships and peer influence [71], school experiences and education [72], living environment and community context [73], social discrimination and stigma [74], gender and sexual identity-based inequalities [75], access to healthcare and psychological support services [76] are often interconnected and influence adolescent behaviors, shaping their choices, habits, and health risks.

Among these determinants, peer-to-peer interactions play a critical role in adolescents’ risk-taking behaviors. Observing peers engage in risky actions, such as reckless driving, increases the likelihood of adolescents adopting similar behaviors [77,78] and negatively impacts cognitive reasoning performance [79].

However, broader “macro-social” factors also significantly influence behaviors. For instance, the impact of poverty on social behaviors and psychological distress during adolescence is well-documented [80]. Children and adolescents from lower-income families often encounter multiple stressors, including food insecurity and unstable housing, which can adversely affect their psychological well-being. Conversely, improving economic conditions can lead to unexpected outcomes, such as the rise in adolescent suicides in Ireland during the early 2000s, which aligns with theories of goal-striving stress [81]. Closely related to poverty, another macro-social factor of interest is social inequality [82,83]. Considering social inequality allows for a better examination of how individual agentic capacities develop in interaction with the structural opportunities and constraints of social contexts, ultimately shaping adolescents’ developmental trajectories [84].

Studies indicate that social inequalities, such as varying income levels and economic opportunities, significantly influence mental health trajectories in younger populations [82]. Additionally, the impact of neighborhood context on mental health is crucial. Areas with high levels of crime, limited access to healthcare and recreational facilities, and fewer positive models can create environments that hinder positive mental health outcomes [85].

Another critical determinant of adolescents’ mental health is access to quality education. It not only provides essential knowledge and skills but also fosters social connections and self-esteem. Research has shown that educational attainment correlates with mental health outcomes, as individuals with higher levels of education generally report lower incidences of anxiety and depression. Conversely, educational disparities can exacerbate issues of social inequality, leading to a cycle where disadvantaged youth are more vulnerable to mental health disorders.

Physical features of the environment further complicate the interplay between several factors on adolescents’ mental health. The effects of pollution and climate change on adolescent behavior and mental health are emerging areas of study. A recent systematic review examined the impact of various pollution dimensions on adolescent mental health [86]. The authors concluded that exposure to air and water pollution was associated with heightened symptoms of depression, generalized anxiety, psychosis, and disruptive or impulse-control disorders. Moreover, exposure to lead and solvents was linked to neurodevelopmental impairments. These findings underscore the significant mental health risks posed by pollution exposure among adolescents.

Connected to these concerns are the anxiety and stress associated with adolescents’ awareness and beliefs regarding pollution and climate change. To what extent can these beliefs act as a source of resilience, and to what degree do they function as risk factors? [87]. Social changes in human populations are highly complex. Traits considered maladaptive in “stable” environments may become adaptive in rapidly changing circumstances, especially in the context of shifting environmental conditions such as altered biorhythms, light and noise pollution [88]. These considerations are particularly relevant to adolescence, a life stage characterized by heightened sensitivity to novelty [89].

### 4.3. Focal Point 3: Discrimination, Social Stigma and Adolescents’ Beliefs About Mental Health

As is well known, discrimination is the tangible manifestation of exclusion, denial of rights, or unfair treatment. At its core lies stigma, a social and cultural process that reinforces stereotypes and prejudices, shaping perceptions of certain individuals as inferior or different. Many adolescents experience several kinds of discrimination and social stigma, such as ethnic [90], gender-based [91], sexual orientation-related [92], weight-related [93], and it may develop mental health issues such as avoidance behaviors, aggression, suicide behaviors or substance use as coping mechanisms.

The stigmatization of young people with mental health disorders is a pervasive and disabling issue, affecting both children and adults. It was pointed out that, while some variation can be observed based on diagnosis and gender, stigmatization is generally unaffected by labeling and that self-stigmatization among youths resulted in increased secrecy and a tendency to avoid interventions [94].

To fully understand the phenomenon and develop effective preventive interventions, it is crucial to explore not only adolescents’ beliefs about mental health but also those of parents and teachers. Although there is a considerable amount of literature addressing public perceptions of mental illness [95,96], particularly within the context of cultural differences [97], recent studies specifically focusing on adolescence are relatively scarce [98,99]. Addressing this gap is vital, as distorted beliefs can lead to stigma, self-stigma, discrimination, social isolation, and delays in seeking help.

Furthermore, considering the dialectical relationship in which adolescents usually shape their beliefs through interactions with adults, examining the differences in perspectives among adolescents, parents, and educators is crucial. A particularly effective and validated method involves using briefcase vignettes. These vignettes depict hypothetical individuals exhibiting symptoms typical of psychiatric disorders (e.g., a depressive episode or attention-deficit/hyperactivity disorder) and encourage respondents to express their beliefs regarding the nature of the disorder, potential causes, and recommended interventions [100,101].

### 4.4. Focal Point 4: Online Behaviors, Socialization and Loneliness

In 2022, up to 95% of adolescents aged 13 to 17 used social media platforms, with over a third stating that they are on social media “almost constantly” [102]. These data are also shaped by the COVID-19 pandemic, which has increased their consumption patterns. The Internet and social media platforms represent a rapidly evolving space where adolescents play, have fun, create content, shape, compare, solve school tasks, and define their social interactions. Social media platforms support various verbal and visual communication modes through internet-based networking, fostering social connectivity and facilitating real-time interaction. In this context, online social interactions or merely engaging with social networks can play a significant role in adolescents’ lives, shaping their behaviors and influencing their interpersonal relationships. Depending on individual factors, extensive online exposure to peer influences can heighten susceptibility to both positive and negative social pressures [103,104]. While social networks can serve as a valuable tool for securing social support, accessing targeted support interventions, and engaging in advocacy efforts, they can also expose adolescents to serious risks, such as peer influence regarding sexual behavior, expand exposure to a broader network of sexually experienced individuals [105], and cyberbullying [106].

Adolescents’ developing sense of self drives their desire for autonomy. However, their immaturity and limited self-regulation make them more vulnerable to excessive and maladaptive internet use. Problematic internet use is marked by uncontrolled and excessive engagement with the internet, leading to negative physical and mental consequences. Some researchers refer to this phenomenon as internet addiction or compulsive internet use, which negatively impacts adolescents’ physical and mental health, including poor academic performance, inadequate social adjustment, and emotional issues, such as depression, anxiety, and loneliness [107].

A novel turn of events, beyond internet use, is now social media exposure and its impact on socialization. Many studies have examined the relationship between general social media use and loneliness [107,108]. Loneliness is a subjective emotional state characterized by personal dissatisfaction, often linked to the absence or loss of social connections [109]. Individuals experiencing loneliness are more inclined to engage in online social interactions to alleviate this distressing emotion. Consequently, adolescents experiencing loneliness may be driven by their need for social connection to increasingly rely on the accessible and immersive nature of the internet. This heightened engagement with online interactions can lead to excessive internet use, potentially contributing to the development of problematic internet use. However, findings remain inconclusive regarding whether social networking site use alleviates or exacerbates loneliness, and this phenomenon cannot be overlooked, particularly given the evidence suggesting that loneliness is a strong predictor of early mortality comparable with other well-established factors like cigarette smoking or diabetes [110,111].

Excessive social media use among adolescents may diminish face-to-face social interactions, potentially leading to social isolation, risk of school failure and impacting the regularity of social and behavioral rhythms, including eating, sleeping, aerobic fitness and spending time with friends, with an elevated risk of depression. Prolonged exposure to blue light from screens can negatively impact sleep quality, which is closely linked to depressive symptoms [112].

Furthermore, a critical issue arises that cannot be overlooked. The acquisition and refinement of adolescents’ emotional and social skills could be influenced by fictional environments and scenarios, such as those found in TV series, video games, and virtual spaces [113,114]. Supported by concrete evidence, it has been hypothesized that immersive virtual reality may exert a great influence on the acquisition of social behaviors due to the intense activation of the mirror neuron system during exposure to a virtual environment [115]. As a result, the assimilation of behaviors, such as violent ones, through mediums such as video games could disproportionately emphasize reinforced stimuli without adequately conveying the emotional repercussions that are inherent in direct peer interactions during adolescence.

### 4.5. Focal Point 5: Focus on Pre-Clinical Conditions

Mental health promotion among adolescents needs to identify and focus not only on “full-blown cases” of mental disorders but also on potentially high-risk and pre-clinical conditions. Acknowledging the limitations of existing psychiatric classification and treatment frameworks concerning mental health in young populations and early intervention services, transdiagnostic clinical staging models have emerged as a promising alternative [19]. These models try to extend beyond the linear progression of illness to incorporate broader dimensions of disease expansion, as reflected in the development of mental or physical comorbidities, increasing clinical complexity, or significant alterations in associated biological markers.

Progressive clinical stages are delineated based on conventional symptom profiles that differentiate between “sub-threshold” and “threshold-level” disorders despite both necessitating clinical evaluation and possible intervention. In this way, they focus on positioning individuals along a continuum of illness to enhance treatment selection and deepen the understanding of illness trajectories, including patterns of continuity, discontinuity, and etiopathogenesis.

This approach is particularly relevant for addressing help-seeking behaviors and the mental health challenges prevalent during the critical developmental stages of adolescence, which often represent the peak age range for onset.

Furthermore, for similar reasons previously outlined regarding beliefs about mental illness, the identification of potentially risky and pre-clinical conditions should be explored not only among adolescents but also among their parents and teachers. A previous European multi-center study involving school-age children (but not adolescents) found significant discrepancies between parents, children, and teachers in recognizing pre-clinical conditions. These divergences were particularly pronounced in identifying children with a high probability of experiencing internalizing disorders [116]. Given the natural distancing and independence-seeking behaviors of adolescents, these discrepancies may become even more pronounced during this stage of development [117].

Transdiagnostic clinical staging models are also relevant from a public health perspective. They allow for the distinction between early risk factors (e.g., childhood maltreatment, neurodevelopmental disorders with childhood onset), some of which can be mitigated through broad, population-level health initiatives, and mild clinical states that are unlikely to progress to more severe illness and may benefit from general supportive interventions. Additionally, they should differentiate these from attenuated syndromes, which carry a higher risk of progression and may necessitate prompt active intervention or targeted secondary prevention strategies [118].

Interestingly, and in line with this approach, the concept of “sub-thresholds” could also be considered from an evolutionary standpoint when applied to the broad bipolar spectrum. Understood, in a neo-Kraepelinian framework, the bipolar spectrum encompasses a much wider range than currently acknowledged by prevailing nosographies [119], with particular relevance to adolescence. It has been hypothesized that traits and behaviors associated with hyperactivity, hyper-exploration and novelty-seeking, including, thus, sub-threshold conditions for bipolar disorders, exist on a continuum. This continuum spans from adaptive behaviors, such as those observed in athletes during performance, to nonspecific stress states that may precede the onset of psychopathological disorders, not exclusively bipolar disorders. For instance, the “DYMERS syndrome” [120] illustrates this progression, which can ultimately extend to fully developed bipolar disorders.

Research further suggests that individuals with hyperactivity traits may respond to stressors associated with social crises by exhibiting innovative and transformative behaviors, which can lead to adaptive social outcomes [88,121]. Supporting this hypothesis, a study conducted on healthy, socially well-adjusted individuals participating in a trial to promote well-being through physical exercise [122] revealed that hyperactivity traits, in the absence of psychopathology, were associated with genetic variants typically linked to a bipolar disorder [123]. Ultimately, subthreshold manic symptoms may confer adaptive advantages in specific contexts, particularly when individuals face novel stressors requiring creative problem-solving [88,121].

This perspective challenges the prevailing view that subthreshold symptoms primarily represent vulnerability factors for future psychopathological disorders, instead highlighting their potential for adaptive functionality.

### 4.6. From a Multi-Level Framework to a Matrix of Measures for Research

Rather than creating a “puzzle” where individual pieces are merely fitted together, a multi-level framework on adolescents’ mental health could provide a foundation for examining the dynamic interactions among various social, community, and individual factors. Building on the previously discussed focal domains, we propose considering certain levels of information regarding (1) macro-level data on social and environmental factors in the sample areas, (2) medium-level data on family and community, (3) adolescent-level data on risk and protective factors for mental health.

Even if not exhaustive, this synthesis can enable the development of a matrix of indicators and measures to systematically capture these data and facilitate a holistic understanding of how various factors interact. This matrix, in turn, could allow multilevel analyses to consider the relationships between macro-, medium-, and micro-level factors chosen as determinants, as well as between each level on one side and different outcomes on the other. Furthermore, the impact of each level should be controlled for the impact of the other levels (between-level analysis), and the indicators within each level should be controlled for others at the same level.

“The Rainbow model” [124,125,126] is a comprehensive, multilevel framework that illustrates how health determinants interact. This model highlights the impact of social and environmental factors on an individual’s risk of illness, capacity for disease prevention, and access to effective treatment. Differently from other models, which often emphasize risk factors predominantly, the “Rainbow model” allows considering health determinants as health-promoting (i.e., salutogenic), protective (i.e., vaccine), or health-damaging risks, depending on what is most appropriate with research hypotheses. At its core, the model positions the individual, whose health is partly shaped by fixed factors such as sex, age, and genetic predisposition. Surrounding this central element are multiple layers of modifiable determinants, including personal lifestyle choices, social and community networks, as well as economic, cultural, and physical environmental factors, all of which interact to shape health trajectories. Drawing inspiration from this model, various authors have proposed and tested several frameworks by multilevel analyses [127,128,129], in which layers are specified before the analysis to define the clusters that the analyses are performed within, usually (1) micro (individual), (2) medium (community), and (3) macro (sample area). Each layer includes specific indicators and measures, eventually declared as mediators.

We believe that a similar approach can be adopted to structure a multi-level framework, along with a corresponding matrix of indicators and measures. It can be used across multiple domains, including empirical research, public health, clinical care, and policy development. This structured approach is needed to operationalize complex determinants of adolescent mental health and to guide both the construction of explanatory models and the design of targeted and effective interventions.

### 4.7. Practical Implications

The framework presented in this study delineates modifiable targets for early intervention and prevention, such as strengthening supportive family and peer environments, enhancing emotional regulation and cognitive flexibility, addressing socioeconomic inequalities, and mitigating exposure to adverse contexts like discrimination or online victimization. It further promotes the ecological alignment of interventions, ranging from school-based programs that address peer dynamics to community initiatives targeting structural determinants and individualized clinical approaches focused on self-regulation and distress management. This multilevel orientation supports a more precise and effective allocation of resources, informing integrative strategies across healthcare, education, and social sectors. Importantly, the factors proposed and discussed within the framework are illustrative rather than exhaustive, and both the domains of interest in adolescent mental health and other components of the framework may be progressively refined and adapted over time.

### 4.8. Constraints and Challenges

Despite its strengths, the present Delphi study is limited by the exclusive inclusion of professionals in the panel, without the involvement of adolescents as participants in either round. This lack of youth representation may restrict the ecological validity of the proposed framework and limit its applicability in informing interventions that are truly responsive to adolescents’ lived experiences. Further research should prioritize the integration of adolescent perspectives, particularly during validation phases, to enhance the relevance and impact of the framework. Furthermore, complementary approaches, such as importance-ranking evaluation within Delphi consensus processes, are warranted to explicitly prioritize focal determinants of adolescent mental health and to better guide decision-making for targeted preventive actions.

Beyond these limitations, applying a multilevel framework entails several methodological and practical challenges. First, collecting and integrating reliable data across macro-, medium-, and micro-levels requires coordinated efforts, institutional collaboration, and access to diverse data sources. Second, multilevel statistical modeling involves complex nested data structures and demands adequate sample sizes, appropriate analytical techniques, and specialized software. Third, although the framework is intended to be flexible and scalable, its application must be culturally and contextually adapted to avoid overgeneralizations or inappropriate inferences. Lastly, successful implementation relies on effective interdisciplinary cooperation, which may be hindered by differences in terminology, objectives, and methodological traditions across disciplines.

## 5. Conclusions

In this study, we developed an integrative and multilevel framework on adolescents’ mental health through a two-round Delphi methodology with an international, multidisciplinary experts’ panel. This framework, focused on five focal domains, holistically considers biological, psychosocial, and environmental factors related to positive mental health, psychosocial distress, and other pre-clinical conditions among adolescents. It also facilitates a deeper understanding and exploration of the interaction between risk and protective factors in adolescent mental health from an evolutionary standpoint.

The framework is designed to potentially cluster specific indicators through a multi-level matrix, useful for conducting multilevel analyses to examine the relationships between macro-, medium-, and micro-level factors selected as determinants, as well as between each level and various outcomes. Its flexibility allows for adaptation in diverse socio-cultural contexts, making it a valuable tool for both global and local action. Future research employing this framework on adolescent mental health can develop and test specific hypotheses regarding the role of various mental health determinants among adolescents, examining their impact on different mental health outcomes, and establishing a strong foundation for targeted and effective preventive interventions.

## Figures and Tables

**Table 1:** Structure of the initial draft version.

Structure	Item	
	Title	An evolutionary preamble towards a shared conceptual framework to understand adolescent mental health and well-being.
	Paragraphs	
Background	1	The Weight of the Issue.
	2	Limitations of Current Approaches.
Focal domain 1	3	The Social Brain in Adolescence and Social Functioning.
	4	The Role of Social and Physical Environments in Neurodevelopment.
	5	Status, Social Roles and well-being in adolescence.
	6	Attachment Avoidance and Epigenetic Modifications.
Focal domain 2	7	The Role of Macro-Social and Environmental Factors.
Focal domain 3	8	Exploring Adolescents’ Beliefs: Can Stigma Exacerbate the Impact?
Focal domain 4	9	The Influence of Peers, Online Behavior, Leisure Activities, and Social Rhythms.
Focal domain 5	10	Focus on pre-clinical conditions.
Multi-level matrix	11	From a Shared Conceptual Framework to a Matrix of Measures for Research.

**Table 2:** Summary of the Panel’ members responses—Round 1.

		Paragraphs of the Initial Draft Version										
Panel Member	Title of the Initial Draft Version	1	2	3	4	5	6	7	8	9	10	11
1												
2												
3												
4												
5												
6												
7												
8												
9												
10												
11												
12												

Legend: 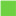 5/5 yes, 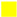 4/5 yes, 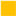 3/5 yes, 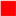 ≤2 yes.

**Table 3:** Consensus index about categories and items—Round 1.

	Item	Relevance	Consistency	Clarity	Easy of Understanding	Updating	Total Score	Consensus Index
**Initial draft version**	Title	12/12	9/12	10/12	9/12	11/12	51/60	0.85
	1 Paragraph	12/12	8/12	9/12	10/12	11/12	50/60	0.83
	2 Paragraph	11/12	10/12	8/12	10/12	8/12	47/60	0.78
	3 Paragraph	11/12	9/12	9/12	11/12	8/12	48/60	0.8
	4 Paragraph	10/12	9/12	9/12	11/12	9/12	48/60	0.8
	5 Paragraph	10/12	9/12	7/12	9/12	8/12	43/60	0.72
	6 Paragraph	8/12	9/12	10/12	11/12	9/12	47/60	0.78
	7 Paragraph	10/12	9/12	9/12	10/12	8/12	46/60	0.77
	8 Paragraph	10/12	6/12	9/12	11/12	10/12	46/60	0.77
	9 Paragraph	10/12	8/12	9/12	11/12	8/12	46/60	0.77
	10 Paragraph	10/12	9/12	9/12	11/12	8/12	47/60	0.78
	11 Paragraph	10/12	8/12	10/12	10/12	8/12	46/60	0.77
	Total score	124/144	103/144	108/144	124/144	106/144	565/720	0.78
	Consensus index	0.86	0.71	0.75	0.86	0.74	0.78	

**Table 4:** Summary of the Panel’ members responses—Round 2.

	New Version of the Draft After Round 1							
Panel Member	Relevant	Consistent	Clear	Easy to Understand	Up to Date	Consistent with the Changes You Suggested	Improved When Compared to the First Version	Suitable for Publication
1								
2								
3								
4								
5	/	/	/	/	/	/	/	/
6								
7								
8								
9								
10								
11								
12								

Legend: 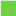 yes, 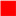 no.

**Table 5:** Consensus index about categories and item—2 Round.

Item	Relevance	Consistency	Clarity	Easy of Understanding	Updating	Consistency with the Changes You Suggested	Improvement When Compared to the First Version	Suitability for Publication	Total Score	Consensus Index
New version of the draft after Round 1	11/11	10/11	11/11	11/11	10/11	11/11	11/11	9/11	84/88	0.95
Consensus index	1.0	0.91	1.0	1.0	0.91	1.0	1.0	0.82	0.95	

**Table 6:** Summary of the main revisions to the initial draft version.

Section		Main Revisions After the Round 1	Main Revisions After the Round 2
Title		The study design has been explicitly stated in the title.	It has been clarified that this is an international Delphi study.
Abstract			It has been added.
1. Introduction	The Weight of the Issue	More specific epidemiological data have been included. The section on suicide has been further elaborated. The bibliography has been updated.	Additional epidemiological considerations regarding gender differences and the prevalence of mental disorders have been incorporated into the manuscript.
	Limitations of Current Approaches on adolescents’ mental health	The section on categorical and dimensional models has been further elaborated. The utility of the evolutionary approach to adolescent mental health in capturing the interplay between risk and protective factors has been explicitly stated.	The importance of the interrelationship between macro-social factors and adolescents’ mental health outcomes has been introduced, as well as to the protective role of factors such as social support, healthy diet, physical activity, and regular sleep habits. Additionally, a statement about the ecological model of health has been incorporated. Additional bibliography has been introduced.
	Proposed Focal Points and Aims	The five focal points have been separated into a dedicated sub-paragraph. They have been more specifically defined as the basis to develop the framework and described. The study objective has been explicitly stated.	The importance of a multidisciplinary approach for the development of a multi-level framework on adolescent mental health has been further clarified.
2. Methods		The Delphi methodology has been introduced and described in detail, specifically: the composition of the research group and the selection of the panel, the first version of the draft that formed the basis of the Delphi study (with a figure), and the rules for the consensus-reaching procedure.	
3. Results		The composition of the panel, as well as the results of the first Delphi round and the achieved consensus indices have been reported (also with tables). The process through which the framework was developed has been presented.	The results of the second Delphi round and the achieved consensus indices have been reported (also with updated tables).
4. Discussion	Focal point 1: The “Social” Brain in Adolescence	It has been divided into four sub-sections. The section on the asynchronous development of the brain has been further elaborated. The section on attachment theory has been more clearly specified. The bibliography has been updated.	A statement has been included with examples of how certain disorders with a strong genetic component, such as ADHD and bipolar disorder, can “shape” the patient’s environmental exposure. The bibliography has been updated.
	Focal point 2: Social determinants, macro-social and environmental factors	The social determinants that most impact adolescent mental health have been explicitly stated. Among these, macro-social and environmental factors have been distinguished. Additional bibliography has been introduced.	It has been further specified how macro-social factors, such as lower family income or limited economic opportunities, may increase exposure to additional stressors such as food insecurity and unstable housing. The role of social factors—such as access to quality education and characteristics of the neighborhood context—on adolescent mental health has also been explicitly addressed. Additional bibliography has been introduced.
	Focal point 3: Discrimination, social stigma and adolescents’ beliefs about mental health	The concept of discrimination and its relationship with stigma phenomenon has been introduced. The main sources of discrimination among adolescents have been described. The concept of stigmatization and self-stigmatization of adolescents with mental health problems has been more clearly specified. Additional bibliography has been introduced.	
	Focal point 4: Online behaviors, socialization and loneliness	A substantial revision has been made regarding the definition and description of online behaviors, particularly related to the use of social media platforms, associated risks, and problematic internet use. The relationship between social networking site use and the phenomenon of loneliness has been more clearly defined. Additional bibliography has been added, especially supporting the relationship between the acquisition of emotional and social skills through fictional environments (i.e., virtual ones).	
	Focal point 5: Focus on Pre-Clinical Conditions	A section on transdiagnostic clinical staging models has been introduced. The part on “sub-threshold” and “threshold-level” disorders has been more clearly explained. In this way, the evolutionary approach to the bipolar spectrum serves as an example for approaching adolescent mental health from a pre-clinical perspective (i.e., regarding hyperactivity, hyper-exploration and novelty-seeking). Additional bibliography has been introduced.	
	From a Multi-level Framework to a Matrix of Measures for Research	A substantial revision has been made regarding how to use the framework through a multi-level indicators matrix. An anchoring to the “Rainbow model” has been proposed, allowing for multi-level analysis to test specific research hypotheses developed based on the framework. Additional bibliography has been introduced.	The areas to be considered in the construction of the matrix have been more clearly defined for macro-level data on social and environmental factors, medium-level data on family and community, and adolescent-level data on risk and protective factors for mental health.
5. Conclusion		Some conclusions have been added, summarizing the main contents of the study.	

## Data Availability

During this study, no new data were created.

## References

[1] Hasin D.S., Fenton M.C., Weissman M.M., Tsuang M.T., Tohen M., Jones P.B. (2011). Epidemiology of Depressive Disorders. Textbook in Psychiatric Epidemiology.

[2] Blakemore S.J. (2019). Adolescence and mental health. Lancet.

[3] Vijayakumar N., Whittle S. (2023). A Systematic Review into the Role of Pubertal Timing and the Social Environment in Adolescent Mental Health Problems. Clin. Psychol. Rev..

[4] Broughton J.M. (1981). The divided self in adolescence. Hum. Dev..

[5] Dalsgaard S., Thorsteinsson E., Trabjerg B.B., Schullehner J., Plana-Ripoll O., Brikell I., Wimberley T., Thygesen M., Madsen K.B., Timmerman A. (2020). Incidence Rates and Cumulative Incidences of the Full Spectrum of Diagnosed Mental Disorders in Childhood and Adolescence. JAMA Psychiatry.

[6] Samji H., Wu J., Ladak A., Vossen C., Stewart E., Dove N., Long D., Snell G. (2022). Review: Mental Health Impacts of the COVID-19 Pandemic on Children and Youth—A Systematic Review. Child Adolesc. Ment. Health.

[7] Erskine H.E., Baxter A.J., Patton G., Moffitt T.E., Patel V., Whiteford H.A., Scott J.G. (2017). The global coverage of prevalence data for mental disorders in children and adolescents. Epidemiol. Psychiatr. Sci..

[8] Solmi M., Radua J., Stubbs B., Ricca V., Moretti D., Busatta D., Carvalho A.F., Dragioti E., Favaro A., Monteleone A.M. (2021). Risk Factors for Eating Disorders: An Umbrella Review of Published Meta-Analyses. Braz. J. Psychiatry.

[9] Paladini G., Sciurpa E., Onorati R., Elhadidy H.S.M.A., Giacomini G., Mamo C., Borraccino A. (2023). Gender and Age Influence on Emergency Department Visits for Non-Suicidal Self-Injuries in School Aged Children in Italy: An 11 Years Retrospective Cross-Sectional Study. Int. J. Public Health.

[10] Senapati R.E., Jena S., Parida J., Panda A., Patra P.K., Pati S., Kaur H., Acharya S.K. (2024). The Patterns, Trends and Major Risk Factors of Suicide among Indian Adolescents—A Scoping Review. BMC Psychiatry.

[11] Mehanović E., Rosso G., Cuomo G.L., Diecidue R., Maina G., Costa G., Vigna-Taglianti F. (2024). Risk Factors for Suicide Reattempt among Adolescents and Young Adults: The Role of Psychiatric Disorders. Psychiatr. Q..

[12] Douglas R.D., Alli J.O., Gaylord-Harden N., Opara I., Gilreath T. (2025). Examining the integrated model of the interpersonal-psychological theory of suicide and intersectionality theory among Black male adolescents. Suicide Life Threat. Behav..

[13] Xu M., Rosario-Williams B., Kline E.A., Miranda R. (2022). Social Cognitive Mechanisms between Psychological Maltreatment and Adolescent Suicide Ideation: Race/Ethnicity and Gender as Moderators. Psychol. Violence.

[14] Chu J., Goldblum P., Floyd R., Bongar B. (2020). The cultural theory and model of suicide. Appl. Prev. Psychol..

[15] Hua L.L., Lee J., Rahmandar M.H., Sigel E.J. (2024). Committee on adolescence; council on injury, violence, and poison prevention. Suicide and Suicide Risk in Adolescents. Pediatrics.

[16] Vander Weele T. (2023). Why Are Young People So Miserable?. Harv. Gazette.

[17] Trong Dam V.A., Do H.N., Thi Vu T.B., Vu K.L., Do H.M., Thi Nguyen N.T., Nguyen T.T., Thi Vu T.M., Thi Nguyen T.P., Auquier P. (2023). Associations between Parent-Child Relationship, Self-Esteem, and Resilience with Life Satisfaction and Mental Wellbeing of Adolescents. Front. Public Health.

[18] Carballo J.J., Llorente C., Kehrmann L., Flamarique I., Zuddas A., Purper-Ouakil D., Hoekstra P.J., Coghill D., Schulze U.M.E., Dittmann R.W. (2020). Psychosocial risk factors for suicidality in children and adolescents. Eur. Child Adolesc. Psychiatry.

[19] Shah J.L., Scott J., McGorry P.D., Cross S.P.M., Keshavan M.S., Nelson B., Wood S.J., Marwaha S., Yung A.R., Scott E.M. (2020). Transdiagnostic Clinical Staging in Youth Mental Health: A First International Consensus Statement. World Psychiatry.

[20] Marchini S., Reis J., Ben-Shaool E., Delhaye M., Kornreich C., Nicolis H., Slama H., Leys C., Delvenne V. (2023). Dimensional model on how familial vulnerability and environmental factors impact transitional age youth psychopathology: The Transition_psy study. Front. Psychiatry.

[21] Ingram R.E., Luxton D.D., Hankin B.L., Abela J.R.Z. (2005). Vulnerability-stress models. Development of Psychopathology: A Vulnerability-Stress Perspective.

[22] Quaedflieg C.W.E.M., Smeets T., Gellman M.D. (2020). Stress Vulnerability Models. Encyclopedia of Behavioral Medicine.

[23] Brown S.A., Jernigan T.L., Dowling G.J. (2023). The adolescent brain cognitive development study. Health Psychol..

[24] Masten A.S., Lucke C.M., Nelson K.M., Stallworthy I.C. (2021). Resilience in Development and Psychopathology: Multisystem Perspectives. Annu. Rev. Clin. Psychol..

[25] Berkman L.F., Glass T., Berkman L.F., Kawachi I. (2000). Social integration, social networks, social support and health. Social Epidemiology.

[26] Rodriguez-Ayllon M., Cadenas-Sánchez C., Estévez-López F., Muñoz N.E., Mora-Gonzalez J., Migueles J.H., Molina-García P., Henriksson H., Mena-Molina A., Martínez-Vizcaíno V. (2019). Role of Physical Activity and Sedentary Behavior in the Mental Health of Preschoolers, Children and Adolescents: A Systematic Review and Meta-Analysis. Sports Med..

[27] Recchia F., Bernal J.D.K., Fong D.Y., Wong S.H.S., Chung P.K., Chan D.K.C., Capio C.M., Yu C.C.W., Wong S.W.S., Sit C.H.P. (2023). Physical Activity Interventions to Alleviate Depressive Symptoms in Children and Adolescents: A Systematic Review and Meta-Analysis. JAMA Pediatr..

[28] Camprodon-Boadas P., Gil-Dominguez A., De la Serna E., Sugranyes G., Lázaro I., Baeza I. (2025). Mediterranean Diet and Mental Health in Children and Adolescents: A Systematic Review. Nutr. Rev..

[29] Da Silva L.E.M., Costa P.R.F., Brito Beck da Silva Magalhães K., Cunha C.M., Pinheiro de Oliveira Alves W., Miranda Pereira E., de Santana M.L.P. (2025). Dietary Pattern and Depressive Outcomes in Children and Adolescents: Systematic Review and Meta-analysis of Observational Studies. Nutr. Rev..

[30] Cheung F.T.W., Li X., Hui T.K., Chan N.Y., Chan J.W., Wing Y.K., Li S.X. (2023). Circadian preference and mental health outcomes in youth: A systematic review and meta-analysis. Sleep Med. Rev..

[31] Hawley P.H. (2011). The evolution of adolescence and the adolescence of evolution: The coming of age of humans and the theory about the forces that made them. J. Res. Adolesc..

[32] Weisfeld G.E., Goetz S.M.M., Zilioli S. (2020). Evolutionary Perspective on Adolescence. Encycl. Child Adolesc. Dev..

[33] Bronfenbrenner U. (1979). The Ecology of Human Development: Experiments by Nature and Design.

[34] Fink A., Kosecoff J., Chassin M., Brook R.H. (1984). Consensus methods: Characteristics and guidelines for use. Am. J. Public Health.

[35] Jünger S., Payne S.A., Brine J., Radbruch L., Brearley S.G. (2017). Guidance on Conducting and REporting DElphi Studies (CREDES) in palliative care: Recommendations based on a methodological systematic review. Palliat. Med..

[36] Schifano J., Niederberger M. (2025). How Delphi Studies in the Health Sciences Find Consensus: A Scoping Review. Syst. Rev..

[37] Giedd J.N., Rapoport J.L. (2010). Structural MRI of pediatric brain development: What have we learned and where are we going?. Neuron.

[38] Baker S.T., Lubman D.I., Yücel M., Allen N.B., Whittle S., Fulcher B.D., Zalesky A., Fornito A. (2015). Developmental changes in brain network hub connectivity in late adolescence. J. Neurosci..

[39] Oyefiade A.A., Ameis S., Lerch J.P., Rockel C., Szulc K.U., Scantlebury N., Decker A., Jefferson J., Spichak S., Mabbott D.J. (2018). Development of Short-Range White Matter in Healthy Children and Adolescents. Hum. Brain Mapp..

[40] Taylor B.K., Heinrichs-Graham E., Eastman J.A., Frenzel M.R., Wang Y.P., Calhoun V.D., Stephen J.M., Wilson T.W. (2022). Longitudinal Changes in the Neural Oscillatory Dynamics Underlying Abstract Reasoning in Children and Adolescents. Neuroimage.

[41] Erdoğan Ö. (2023). A system approach to the self: Interpretive phenomenological analysis. Heliyon.

[42] Casey B.J., Getz S., Galvan A. (2008). The adolescent brain. Dev. Rev..

[43] Faraone S.V., Banaschewski T., Coghill D., Zheng Y., Biederman J., Bellgrove M.A., Newcorn J.H., Gignac M., Al Saud N.M., Manor I. (2021). The World Federation of ADHD International Consensus Statement: 208 Evidence-based conclusions about the disorder. Neurosci. Biobehav. Rev..

[44] Singh M.K., Post R.M., Miklowitz D.J., Birmaher B., Youngstrom E., Goldstein B., Soutullo C., Axelson D., Chang K.D., DelBello M.P. (2021). A Commentary on Youth Onset Bipolar Disorder. Bipolar Disord..

[45] Schmitt A., Falkai P., Papiol S. (2023). Neurodevelopmental Disturbances in Schizophrenia: Evidence from Genetic and Environmental Factors. J. Neural Transm..

[46] Cascino G., Monteleone A.M. (2024). Early traumatic experiences and the hypothalamus–pituitary–adrenal axis in people with eating disorders: A narrative review. Psychoneuroendocrinology.

[47] Dekel S., Papadakis J.E., Quagliarini B., Pham C.T., Pacheco-Barrios K., Hughes F., Jagodnik K.M., Nandru R. (2024). Preventing posttraumatic stress disorder following childbirth: A systematic review and meta-analysis. Am. J. Obstet. Gynecol..

[48] Porter C., Palmier-Claus J., Branitsky A., Mansell W., Warwick H., Varese F. (2020). Childhood Adversity and Borderline Personality Disorder: A Meta-Analysis. Acta Psychiatr. Scand..

[49] Sancassiani F., Machado S., Ruggiero V., Cacace E., Carmassi C., Gesi C., Dell’Osso L., Carta M.G. (2017). The management of fibromyalgia from a psychosomatic perspective: An overview. Int. Rev. Psychiatry.

[50] Pasquaretta C., Levé M., Claidière N., van de Waal E., Whiten A., MacIntosh A.J.J., Pelé M., Bergstrom M.L., Borgeaud C., Brosnan S.F. (2014). Social Networks in Primates: Smart and Tolerant Species Have More Efficient Networks. Sci. Rep..

[51] Noonan M.P., Sallet J., Mars R.B., Neubert F.X., O’Reilly J.X., Andersson J.L., Mitchell A.S., Bell A.H., Miller K.L., Rushworth M.F.S. (2014). A Neural Circuit Covarying with Social Hierarchy in Macaques. PLoS Biol..

[52] Von Der Heide R., Vyas G., Olson I.R. (2014). The Social Network-Network: Size Is Predicted by Brain Structure and Function in the Amygdala and Paralimbic Regions. Soc. Cogn. Affect. Neurosci..

[53] Baltruschat S., Megías-Robles A., Cándido A., Maldonado A., Catena A. (2021). Social and non-social brain areas in risk behaviour: The role of social context. Neuroscience.

[54] Veerareddy A., Fang H., Safari N., Xu P., Krueger F. (2023). Cognitive empathy mediates the relationship between gray matter volume size of dorsomedial prefrontal cortex and social network size: A voxel-based morphometry study. Cortex.

[55] Lamblin M., Murawski C., Whittle S., Fornito A. (2017). Social connectedness, mental health and the adolescent brain. Neurosci. Biobehav. Rev..

[56] Ainsworth M., Blehar M., Waters E., Wall S. (1978). Patterns of Attachment.

[57] Ainsworth M., Bowlby J. (1965). Child Care and the Growth of Love.

[58] Barglow P., Vaughn B.E., Molitor N. (1987). Effects of maternal absence due to employment on the quality of infant-mother attachment in a low-risk sample. Child Dev..

[59] NICHD Early Child Care Research Network (1997). The Effects of Infant Child Care on Infant-Mother Attachment Security: Results of the NICHD Study of Early Child Care. Child Dev..

[60] Sullivan R., Lasley E.N. (2010). Fear in Love: Attachment, Abuse, and the Developing Brain. Cerebrum.

[61] Bisaz R., Sullivan R.M. (2012). Developmental neurobiology of the rat attachment system and its modulation by stress. Behav. Sci..

[62] Ein-Dor T., Verbeke W.J.M.I., Mokry M., Vrtička P. (2018). Epigenetic modification of the oxytocin and glucocorticoid receptor genes is linked to attachment avoidance in young adults. Attach. Hum. Dev..

[63] Moser D.A., Paoloni-Giacobino A., Stenz L., Adouan W., Manini A., Suardi F., Cordero M.I., Vital M., Sancho Rossignol A., Rusconi-Serpa S. (2015). BDNF Methylation and Maternal Brain Activity in a Violence-Related Sample. PLoS ONE.

[64] Moser D.A., Graf S., Glaus J., Urben S., Jouabli S., Pointet Perrizolo V., Suardi F., Robinson J., Rusconi Serpa S., Plessen K.J. (2023). On the complex and dimensional relationship of maternal posttraumatic stress disorder during early childhood and child outcomes at school-age. Eur. Psychiatry.

[65] Borelli J.L., Brugnera A., Zarbo C., Rabboni M., Bondi E., Tasca G.A., Compare A. (2019). Attachment comes of age: Adolescents’ narrative coherence and reflective functioning predict well-being in emerging adulthood. Attach. Hum. Dev..

[66] Kirkbride J.B., Anglin D.M., Colman I., Dykxhoorn J., Jones P.B., Patalay P., Pitman A., Soneson E., Steare T., Wright T. (2024). The social determinants of mental health and disorder: Evidence, prevention and recommendations. World Psychiatry.

[67] Reiss F. (2013). Socioeconomic Inequalities and Mental Health Problems in Children and Adolescents: A Systematic Review. Soc. Sci. Med..

[68] Smeeth D., Ecker S., Chervova O., McEwen F., Karam E., Beck S., Pluess M. (2025). War Exposure and DNA Methylation in Syrian Refugee Children and Adolescents. JAMA Psychiatry.

[69] Eckstein K., Crocetti E. (2021). The impact of migration on child and adolescent development: The role of socialization experiences in family and school. New Dir. Child Adolesc. Dev..

[70] Yap M.B., Pilkington P.D., Ryan S.M., Jorm A.F. (2014). Parental Factors Associated with Depression and Anxiety in Young People: A Systematic Review and Meta-Analysis. J. Affect. Disord..

[71] McPherson K.E., Kerr S., McGee E., Morgan A., Cheater F.M., McLean J., Egan J. (2014). The association between social capital and mental health and behavioural problems in children and adolescents: An integrative systematic review. BMC Psychol..

[72] Ye Z., Wu D., He X., Ma Q., Peng J., Mao G., Feng L., Tong Y. (2023). Meta-Analysis of the Relationship between Bullying and Depressive Symptoms in Children and Adolescents. BMC Psychiatry.

[73] Poulain T., Sobek C., Ludwig J., Igel U., Grande G., Ott V., Kiess W., Körner A., Vogel M. (2020). Associations of Green Spaces and Streets in the Living Environment with Outdoor Activity, Media Use, Overweight/Obesity and Emotional Wellbeing in Children and Adolescents. Int. J. Environ. Res. Public Health.

[74] Schmitt M.T., Branscombe N.R., Postmes T., Garcia A. (2014). The Consequences of Perceived Discrimination for Psychological Well-Being: A Meta-Analytic Review. Psychol. Bull..

[75] Marquez J., Humphrey N., Black L., Cutts M., Khanna D. (2023). Gender and sexual identity-based inequalities in adolescent wellbeing: Findings from the #BeeWell Study. BMC Public Health.

[76] Aguirre Velasco A., Cruz I.S.S., Billings J., Jimenez M., Rowe S. (2020). What are the barriers, facilitators and interventions targeting help-seeking behaviors for common mental health problems in adolescents? A systematic review. BMC Psychiatry.

[77] Cascio C.N., Carp J., O’Donnell M.B., Tinney F.J., Bingham C.R., Shope J.T., Ouimet M.C., Pradhan A.K., Simons-Morton B.G., Falk E.B. (2015). Buffering social influence: Neural correlates of response inhibition predict driving safety in the presence of a peer. J. Cogn. Neurosci..

[78] Vorobyev V., Kwon M.S., Moe D., Parkkola R., Hämäläinen H. (2015). Risk-Taking Behavior in a Computerized Driving Task: Brain Activation Correlates of Decision-Making, Outcome, and Peer Influence in Male Adolescents. PLoS ONE.

[79] Wolf L.K., Bazargani N., Kilford E.J., Dumontheil I., Blakemore S.-J. (2015). The Audience Effect in Adolescence Depends on Who’s Looking Over Your Shoulder. J. Adolesc..

[80] Yoshikawa H., Aber J.L., Beardslee W.R. (2012). The Effects of Poverty on the Mental, Emotional, and Behavioral Health of Children and Youth: Implications for Prevention. Am. Psychol..

[81] European Commission (2004). The State of Mental Health in the European Union.

[82] Marmot M., Bell R. (2016). Social inequalities in health: A proper concern of epidemiology. Ann. Epidemiol..

[83] Pickett K.E., Wilkinson R.G. (2015). Income Inequality and Health: A Causal Review. Soc. Sci. Med..

[84] Buchmann M., Steinhoff A. (2017). Social Inequality, Life Course Transitions, and Adolescent Development: Introduction to the Special Issue. J. Youth Adolesc..

[85] Patel V., Kleinman A. (2003). Poverty and Common Mental Disorders in Developing Countries. Bull. World Health Organ..

[86] Theron L.C., Abreu-Villaça Y., Augusto-Oliveira M., Brennan C., Crespo-Lopez M.E., de Paula Arrifano G., Glazer L., Gwata N., Lin L., Mareschal I. (2022). A systematic review of the mental health risks and resilience among pollution-exposed adolescents. J. Psychiatr. Res..

[87] Thomas I., Martin A., Wicker A., Benoit L. (2022). Understanding youths’ concerns about climate change: A binational qualitative study of ecological burden and resilience. Child Adolesc. Psychiatry Ment. Health.

[88] Carta M.G., Preti A., Akiskal H.S. (2018). Coping with the new era: Noise and light pollution, hyperactivity and steroid hormones. Towards an evolutionary view of bipolar disorders. Clin. Pract. Epidemiol. Ment. Health.

[89] Del Giacco A.C., Jones S.A., Morales A.M., Kliamovich D., Nagel B.J. (2022). Adolescent novelty seeking is associated with greater ventral striatal and prefrontal brain response during evaluation of risk and reward. Cogn. Affect. Behav. Neurosci..

[90] Yip T., Wang Y., Mootoo C., Mirpuri S. (2019). Moderating the Association between Discrimination and Adjustment: A Meta-Analysis of Ethnic/Racial Identity. Dev. Psychol..

[91] Brown C.S., Stone E.A. (2016). Gender Stereotypes and Discrimination: How Sexism Impacts Development. Adv. Child Dev. Behav..

[92] Nagata J.M., Wong J.H., Helmer C.K., Domingue S.K., Shim J.E., Al-Shoaibi A. (2024). Sexual Orientation Discrimination in Early Adolescents. JAMA Netw. Open.

[93] Nagata J.M., Helmer C.K., Wong J.H., Domingue S.K., Shim J.E., Al-Shoaibi A.A.A. (2024). Prevalence and Sociodemographic Associations with Weight Discrimination in Early Adolescents. Prev. Med. Rep..

[94] Kaushik A., Kostaki E., Kyriakopoulos M. (2016). The stigma of mental illness in children and adolescents: A systematic review. Psychiatry Res..

[95] Schomerus G., Schindler S., Baumann E., Angermeyer M.C. (2023). Changes in continuum beliefs for depression and schizophrenia in the general population 2011–2020: A widening gap. Soc. Psychiatry Psychiatr. Epidemiol..

[96] Angermeyer M.C., Schomerus G. (2017). State of the art of population-based attitude research on mental health: A systematic review. Epidemiol. Psychiatr. Sci..

[97] Angermeyer M.C., Carta M.G., Ghachem R., Matschinger H., Millier A., Refai T., Schomerus G., Toumi M. (2020). Cultural variations in public beliefs about mental disorders: A comparison between Tunisia and Germany. Clin. Pract. Epidemiol. Ment. Health.

[98] Pinder J.B., Ahuvia I.L., Chen S., Schleider J.L. (2024). Beliefs about depression relate to active and avoidant coping in high-symptom adolescents. J. Affect. Disord..

[99] Ahuvia I.L., Fox K.R., Schleider J.L. (2023). Adolescents’ beliefs about what symptoms constitute depression: Are more expansive definitions helpful or harmful?. SSM Ment. Health.

[100] Angermeyer M.C., Schindler S., Matschinger H., Baumann E., Schomerus G. (2023). The rise in acceptance of mental health professionals: Help-seeking recommendations of the German public 1990–2020. Epidemiol. Psychiatr. Sci..

[101] Moro M.F., Angermeyer M.C., Matschinger H., Holzinger A., Piras A.P., Cutrano F., Mura G., Carta M.G. (2015). Whom to ask for professional help in case of major depression? Help-seeking recommendations of the Sardinian public. Adm. Policy Ment. Health.

[102] Office of the Surgeon General (OSG) (2023). Social Media and Youth Mental Health: The U.S. Surgeon General’s Advisory.

[103] Angelini F., Marino C., Gini G. (2022). Friendship quality in adolescence: The role of social media features, online social support and e-motions. Curr. Psychol..

[104] Dienlin T., Johannes N. (2020). The impact of digital technology use on adolescent well-being. Dialogues Clin. Neurosci..

[105] Gewirtz O’Brien J., McPherson L., Miller K., Svetaz M.V. (2021). Adolescent Health: Media Use. FP Essent..

[106] Chan T.K.H., Christy M.K.C., Zach W.Y.L. (2021). Cyberbullying on social networking sites: A literature review and future research directions. Inf. Manag..

[107] Wang P., Wang X., Gao T., Yuan X., Xing Q., Cheng X., Ming Y., Tian M. (2023). Problematic Internet Use in Early Adolescents: Gender and Loneliness Differences in a Latent Growth Model. Psychol. Res. Behav. Manag..

[108] Pop L.M., Iorga M., Iurcov R. (2022). Body-Esteem, Self-Esteem and Loneliness among Social Media Young Users. Int. J. Environ. Res. Public Health.

[109] Bowirrat A., Elman I., Dennen C.A., Gondré-Lewis M.C., Cadet J.L., Khalsa J., Baron D., Soni D., Gold M.S., McLaughlin T.J. (2023). Neurogenetics and epigenetics of loneliness. Psychol. Res. Behav. Manag..

[110] Holt-Lunstad J., Smith T.B., Layton J.B. (2010). Social relationships and mortality risk: A meta-analytic review. PLoS Med..

[111] Aiello A.E. (2017). Invited commentary: Evolution of social networks, health, and the role of epidemiology. Am. J. Epidemiol..

[112] Gao J., Gao L. (2024). A meta-analysis of prospective cohort studies on screen time and the risk of depression in adolescents. Acta Psychol..

[113] García-Gil M.Á., Revuelta-Domínguez F.-I., Pedrera-Rodríguez M.-I., Guerra-Antequera J. (2024). Exploring Video Game Engagement, Social–Emotional Development, and Adolescent Well-Being for Sustainable Health and Quality Education. Sustainability.

[114] Arias E. (2019). Does media influence social norms? Experimental evidence on the role of common knowledge. Political Sci. Res. Methods.

[115] Carta M.G., Nardi A.E. (2024). Virtual reality, social intelligence, mirror neurons and bipolar spectrum: A new perspective. Braz. J. Psychiatry.

[116] Kovess V., Carta M.G., Pez O., Bitfoi A., Koç C., Goelitz D., Kuijpers R., Lesinskiene S., Mihova Z., Otten R. (2015). The School Children Mental Health in Europe (SCMHE) Project: Design and First Results. Clin. Pract. Epidemiol. Ment. Health.

[117] Cheng S., Keyes K.M., Bitfoi A., Carta M.G., Koç C., Goelitz D., Otten R., Lesinskiene S., Mihova Z., Pez O. (2018). Understanding parent-teacher agreement of the Strengths and Difficulties Questionnaire (SDQ): Comparison across seven European countries. Int. J. Methods Psychiatr. Res..

[118] Iorfino F., Scott E.M., Carpenter J.S., Cross S.P., Hermens D.F., Killedar M., Nichles A., Zmicerevska N., White D., Guastella A.J. (2019). Clinical Stage Transitions in Persons Aged 12 to 25 Years Presenting to Early Intervention Mental Health Services with Anxiety, Mood, and Psychotic Disorders. JAMA Psychiatry.

[119] Carta M.G., Angst J. (2016). Screening for bipolar disorders: A public health issue. J. Affect. Disord..

[120] Carta M.G., Fornaro M., Primavera D., Nardi A.E., Karam E. (2024). Dysregulation of mood, energy, and social rhythms syndrome (DYMERS): A working hypothesis. J. Public Health Res..

[121] Carta M.G., Carpiniello B., Dazzan P., Reda M.A. (2000). Depressive symptoms and occupational role among female groups: A research in a south-east African village. Psychopathology.

[122] Carta M.G., Cossu G., Pintus E., Zaccheddu R., Callia O., Conti G., Pintus M., Aviles Gonzalez C.I., Massidda M.V., Mura G. (2021). Moderate exercise improves cognitive function in healthy elderly people: Results of a randomized controlled trial. Clin. Pract. Epidemiol. Ment. Health.

[123] Kalcev G., Scano A., Orrù G., Primavera D., Cossu G., Nardi A.E., Carta M.G. (2023). Is a Genetic Variant Associated with Bipolar Disorder Frequent in People without Bipolar Disorder but with Characteristics of Hyperactivity and Novelty Seeking?. Clin. Pract. Epidemiol. Ment. Health.

[124] Dahlgren G., Whitehead M. (2021). The Dahlgren-Whitehead model of health determinants: 30 years on and still chasing rainbows. Public Health.

[125] Dahlgren G., Whitehead M. (1991). Policies and strategies to promote social equity in health. Inst. Future Stud..

[126] Dyar O.J., Haglund B.J.A., Melder C., Skillington T., Kristenson M., Sarkadi A. (2022). Rainbows over the world’s public health: Determinants of health models in the past, present, and future. Scand. J. Public Health.

[127] Clench-Aas J., Bergande I., Nes R.B., Holte A. (2021). Trust Buffers Against Reduced Life Satisfaction When Faced with Financial Crisis. Front. Psychol..

[128] Clench-Aas J., Holte A. (2021). Political Trust Influences the Relationship Between Income and Life Satisfaction in Europe: Differential Associations with Trust at National, Community, and Individual Level. Front. Public Health.

[129] Bambra C., Gibson M., Sowden A., Wright K., Whitehead M., Petticrew M. (2010). Tackling the wider social determinants of health and health inequalities: Evidence from systematic reviews. J. Epidemiol. Community Health.

